# A retrospective analysis of Stewart-Treves syndrome in the context of chronic lymphedema^☆^

**DOI:** 10.1016/j.abd.2022.04.011

**Published:** 2023-02-04

**Authors:** Kun Hao, Yuguang Sun, Yan Zhu, Jianfeng Xin, Li Zhang, Bin Li, Wenbin Shen

**Affiliations:** a Department of Lymphatic Surgery, Beijing Shijitan Hospital, Capital Medical University, Beijing, China; b Department of Nuclear Medicine, Beijing Shijitan Hospital, Capital Medical University, Beijing, China; c Department of MR, Beijing Shijitan Hospital, Capital Medical University, Beijing, China

**Keywords:** Lymphangiosarcoma, Lymphedema, Stewart Treves syndrome

## Abstract

**Background:**

stewart-treves syndrome (STS) is an angiosarcoma associated with chronic lymphedema.

**Objectives:**

This article analyses the characteristics of twenty-two patients and proposes active intervention in lymphedema and the early diagnosis of STS.

**Methods:**

Twenty-two patients with STS were diagnosed at the centre over an 11-year period. Clinical manifestations, a series of conventional analyses, and histopathology were used to study these cases retrospectively.

**Results:**

The age range of 22 patients with STS was 15 to 78 years. The main clinical manifestations included multiple skin and subcutaneous nodules and scattered red or purplish-red rashes in the lymphoedematous limbs. These patients often showed clinical symptoms such as lymphedema, weakness, emaciation, pain, mass, lymphadenopathy and so on. The positive rates of ultrasonography, MRI and radionuclide imaging were 66.7% (6/9), 92.3% (12/13) and 18.2% (2/11), respectively. The main points regarding active intervention in lymphedema and early diagnosis of STS were summarized.

**Study limitations:**

Since this was a retrospective study, the main points summarized by the author need to be further quantified in clinical work to guide the diagnosis of this kind of disease more conveniently. In addition, further clinical trials are needed to evaluate the role of lymphedema in the occurrence and development of malignant tumors.

**Conclusions:**

STS can appear in lymphoedematous tissue many years after lymphedema onset. To avoid delays in the diagnosis and therapy of STS, physicians should actively look for signs or symptoms of malignant lymphedema during the follow-up period and promptly manage patients developing problems.

## Introduction

The lymphatic system is a part of both the circulatory and immune systems. The lymphatic system has several critical functions in regulating different components of the homeostasis of the body. As a part of the immune system, the lymph system protects the body from infectious and foreign agents and removes waste products and cellular debris from the interstitium of tissues. Lymphedema can be defined as the abnormal accumulation of protein-rich interstitial fluid that occurs primarily as a consequence of malformation, dysplasia, or acquired disruption of lymphatic circulation. Lymphedema can be distinguished as primary and secondary forms. Primary lymphedema is caused by an inherent defect in the lymphatic vessels or lymph nodes. Secondary lymphedema is the result of various aetiologies, such as trauma, infection, persistent inflammation, venous disease, extreme chronic immobility, malignancy, surgery, and radiation therapy.^1^, ^2^ The lymphoedematous limb has a propensity for bacterial, fungal and viral infections and is at risk for the occurrence and metastasis of malignant tumors,^1^ especially angiosarcoma.^3^, ^4^

stewart-treves syndrome (STS) is an angiosarcoma associated with chronic lymphedema and has a poor prognosis due to the high incidence of local recurrence and distant metastasis, with a median survival rate of only 2.5 years.^5^ Hence, early diagnosis and treatment are very important. However, due to its rarity, neither pathogenesis nor a standard treatment strategy has been established. The aim of this article was to characterize cases of STS treated at the centre and describe their treatments and outcomes to explore future protocols for follow-up of chronic lymphedema and management of STS.

## Materials and methods

### Research subjects

After obtaining institutional review board approval, the authors performed a retrospective review of 22 patients with STS in the Department of Lymphatic Surgery, Beijing Shijitan Hospital, Capital Medical University from May 22, 2007, to December 31, 2018. All patients sought treatment at the centre with lymphedema as the main complaint. The original investigations and treatment for lymphedema were performed, with a referral when the diagnosis of STS was evident.

### Research methods

The clinicopathological features of twenty-two patients were analyzed retrospectively, including clinical manifestations, laboratory tests, ultrasonography, Magnetic Resonance Imaging (MRI), radionuclide imaging and pathological diagnosis. All patients were followed up by telephone.

### Statistical analysis

SPSS ver. 22.0 (SPSS Inc., Chicago, IL, USA) was used for all analyses. Survival was calculated using the Kaplan-Meier method. Cases with unreported survival times were excluded from the survival analysis. Survival time was measured from the time of diagnosis to the time of death or the end of follow-up. A p-value of < 0.05 was considered statistically signiﬁcant.

## Results

### Clinical characteristics

**Figure 1:**
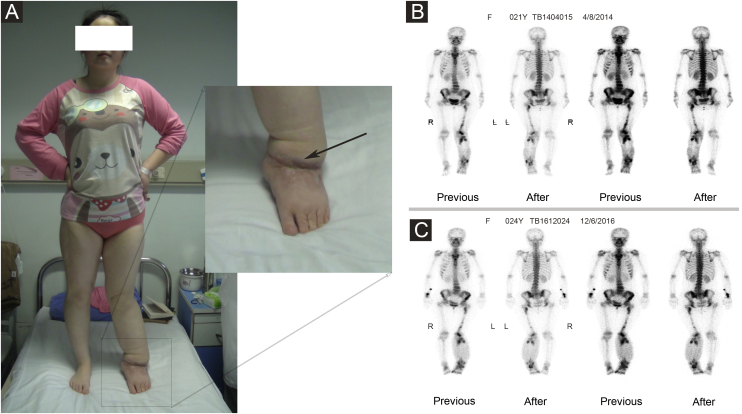
(A) The patient had primary lymphedema of the left lower limb for 21 years. Liposuction and excision of local lesions were performed on the left lower limb. Angiosarcoma of the left lower limb was found by pathology after operation. (B) Bone imaging before operation in 2014. (C) Bone imaging after operation in 2016.

The authors performed a retrospective review of 4676 patients with lymphedema between May 22, 2007, and December 31, 2018. A total of 22 patients with Stewart-Treves syndrome with lymphedema were found. The age range was 15 to 78 years (mean age: 52.7 ± 7.2 years) (Table 1). Three patients were men, and 19 patients were women. The mean duration from lymphedema to STS was 13 years (range: 6 months to 60 years). In all, 12 patients had upper extremity lymphedema after radical mastectomy, 2 patients had lower extremity lymphedema after a cervical cancer operation, 7 patients had primary lower extremity lymphedema (1 patient in Fig. 1), and 1 patient had primary lymphedema of the four extremities and face. Usually, in the area of lymphedema, STS initially appears as multiple, rapidly growing, red-blue macules with surrounding indurations and then becomes plaques of coalescing purple papules with necrotic precincts. Sometimes, the clinical manifestation may have the appearance of a non-resorbing hematoma. As lesions increase, overlapping atrophic skin may ulcerate, which may further lead to infection and bleeding.^6^ These patients also had clinical symptoms such as weakness, emaciation, pain, mass, lymphadenopathy, and so on. The clinical features of each patient are summarized in Table 2.

**Table 1:** Age distribution of 22 patients with Stewart-Treves syndrome.

Age, yr	Nº	Percentage (%)
≤ 20	1	4.5
21‒30	2	9.1
31‒40	2	9.1
41‒50	3	13.6
51‒60	5	22.7
61‒70	6	27.3
71‒80	3	13.6
≤ 35	3	13.6
36‒80	19	86.4

**Table 2:** Clinical features of 22 patients with Stewart-Treves syndrome.

Symptoms and signs	Number of cases	Percentage (%)
STS with upper limb lymphedema (12 cases)		
Upper limb lymphedema	12	100.0
Lymphadenopathy and lump	7	58.3
Ecchymosis and rash	6	50.0
Pain of upper limb	3	25.0
Percolation fluid	3	25.0
Skin redness and high skin temperature	1	8.3
STS with lower limb lymphedema (9 cases)		
Lower limb lymphedema	9	100.0
Lymphadenopathy and lump	6	66.7
Ecchymosis and rash	2	22.2
Pain of lower limb	4	44.4
Percolation fluid	2	22.2
Skin redness and high skin temperature	1	11.1
STS with systemic edema (1 case)		
Swelling of right lower limb, perineal area, left upper limb and left face	1	100.0
Dyskinesia of left upper limb	1	100.0
Pain of upper limb	1	100.0
Lump	1	100.0

### Auxiliary examination

**Figure 2:**
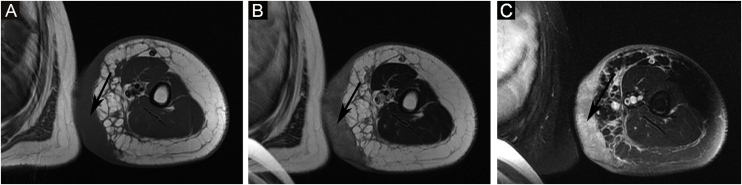
MRI showed nodules distributed on the surface of the skin and subcutaneous tissue; it appeared long T1 and long T2 signals. (A) AXI SE T1. (B) AX T2 FSE. (C) AX fs T2 FSE.

**Figure 3:**
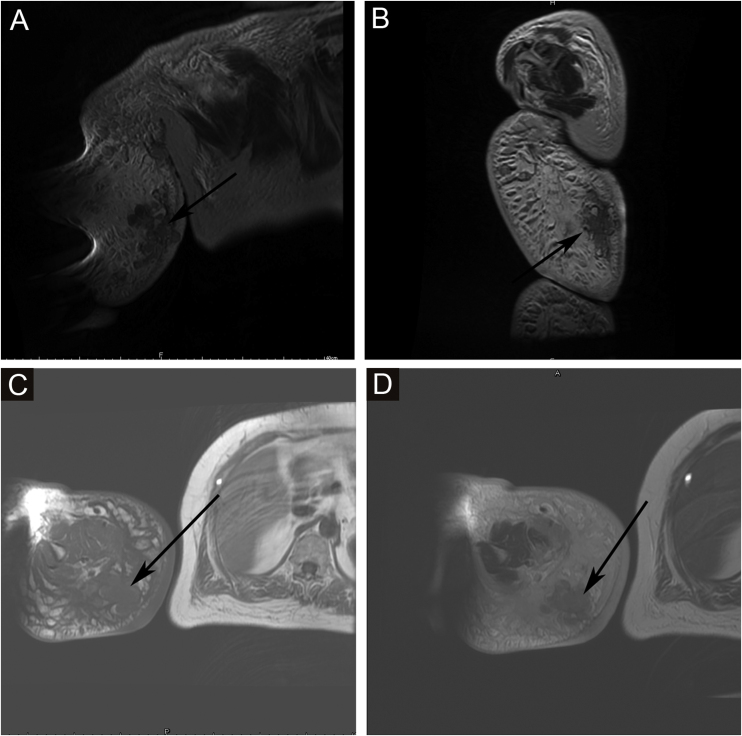
MRI showed nodules distributed on the surface of the skin, subcutaneous tissue and some muscles; it appeared slightly long T1 and short T2 signals. (A) AX fs T2 FSE. (B) AX fs T2 FSE. (C) AXI SE T1. (D) AX T2 FSE.

The proportion of patients with abnormal tumor markers in this study was 31.3% (5/16), including CA153 (1 case), CA199 (1 case), urinary Ig kappa light chain (2 cases), serum β2-microglobulin (2 cases), urinary Ig lambda light chain (1 case), serum Ig kappa light chain (1 case), urinary β2-microglobulin (1 case), AFP (1 case), CA50 (1 case), and serum Ig lambda light chain (1 case). The prevalence of anemia among patients with Stewart-Treves syndrome was 20.0% (5/25). Some suspicious lesions were investigated with imaging examinations. The positive rates of ultrasonography, MRI and radionuclide imaging were 66.7% (6/9), 92.3% (12/13), and 18.2% (2/11), respectively. Ultrasound examination showed that there were multiple masses in the skin and subcutaneous tissue; 1 case appeared hyperechoic and anechoic, 3 cases appeared unevenly hypoechoic, 1 case appeared unevenly hypoechoic and anechoic, and 1 case appeared to have an equal echo. MRI showed nodules distributed on the surface of the skin, subcutaneous tissue, and some muscles; 6 cases appeared with long T1 and long T2 signals (Fig. 2), 1 case appeared with slightly long T1 and short T2 signals (Fig. 3), and 6 cases appeared slightly long T1 signals. Abnormal contrast enhancement was seen on enhanced scanning. Whole-body bone scans (radionuclide bone scans) showed increased bone metabolism (Fig. 1).

### Pathological diagnosis

**Figure 4:**
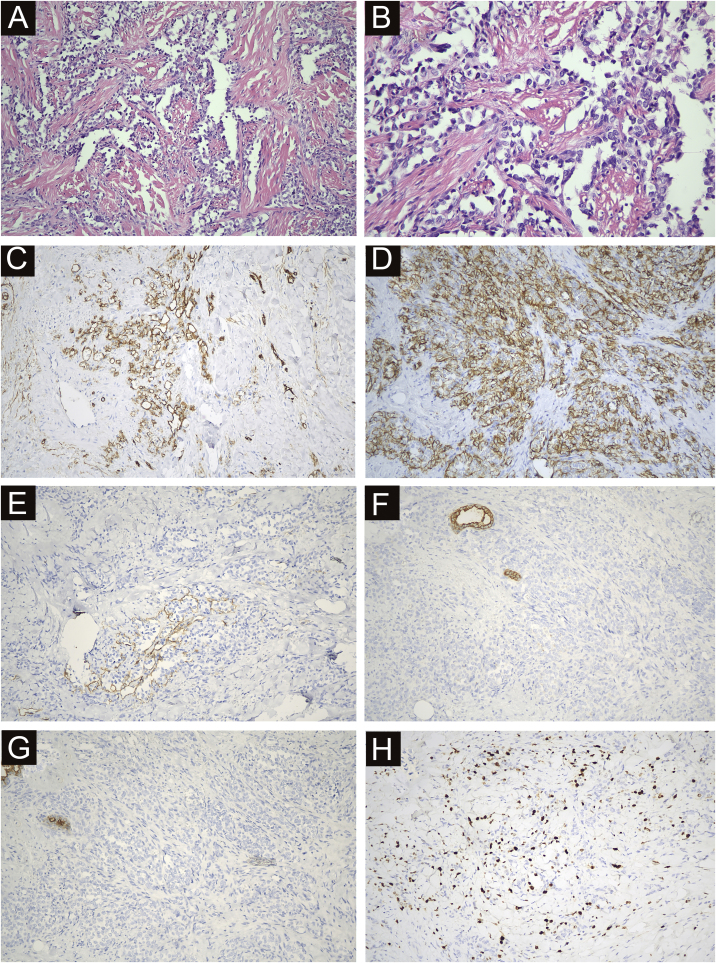
Immunohistochemical and antibody staining of STS. (A) The histopathologic study showed irregular lumens with infiltrative growth in the dermis and subcutaneous tissue, some lumens fused and communicated with each other, and red blood cells could be seen in some lumens. (×200). (B) The epithelium in the lumen was obviously heteromorphic, protruding into the lumen like a tack, and the nucleus was large and darkly stained. (×400). (C) CD34 positive (×200). (D) CD31 positive (×200). (E) D2-40 Locally positive (×200). (F) CK negative (×200). (G) EMA negative (×200). (H) ki67 index 15% (×200).

All 22 cases were confirmed by pathological diagnosis. Tissue samples were taken from the scattered nodules around the diseased area, including skin and subcutaneous tissue. The histopathologic study showed irregular lumens with infiltrative growth in the dermis and subcutaneous tissue, some lumens fused and communicated with each other, and red blood cells could be seen in some lumens. The epithelium in the lumen was obviously heteromorphic, protruding into the lumen like a tack, and the nucleus was large and darkly stained. In some areas, the tumor cells were solid, with obvious nucleoli, mitoses, bleeding and necrosis. Immunohistochemical staining was positive for CD34, CD31 and D2-40 and negative for CK, EMA and HHV-8. The positivity of Ki-67 was counted as 2%‒90% (Fig. 4).

### Treatment and follow-up

**Figure 5:**
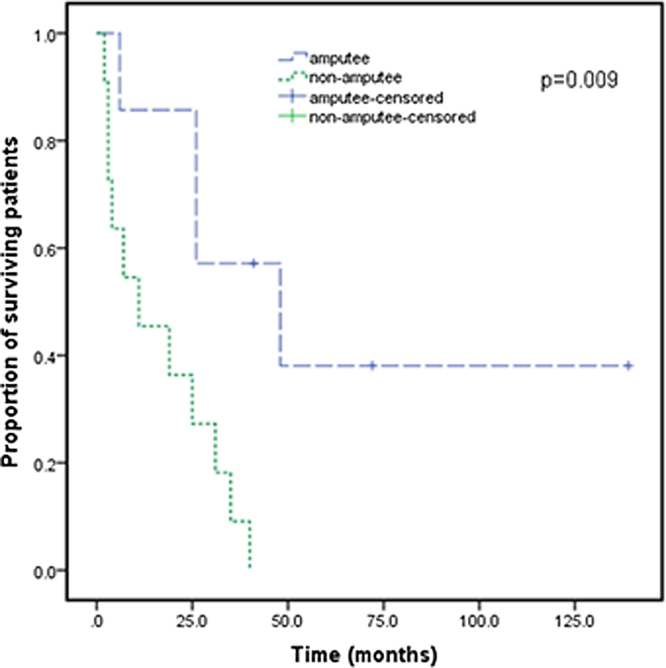
Survival curve for cases of Stewart-Treves syndrome with amputees and without amputees. Survival time was deﬁned as the survival time from diagnosis to the time of death or the end of follow-up. Survival was calculated using Kaplan-Meier analysis. For Stewart-Treves syndrome, the median survival was 25-months, and the 3^rd^ Quartile (Q3) survival was 6-months.

After the patients were diagnosed with Stewart-Treves syndrome in the department, they were transferred to the relevant professional departments for surgery, radiotherapy or chemotherapy. Follow-up data were as follows. There were 12 STS patients with upper extremity lymphedema (Table 3), and 2 patients were lost to follow-up. There were 9 STS patients with lower extremity lymphedema (Table 4), and 4 patients underwent extended resection of lower extremity tumors. They died 2, 3 and 11 months after the operation, and 1 patient was lost to follow-up. For all patients, the median survival was 25 months, and the 3^rd^ Quartile (Q3) survival was 6 months. The survival rate of amputated patients was significantly higher than that of non-amputated patients (p < 0.05) (Fig. 5). There was no significant improvement in survival with radiotherapy, chemotherapy or extended resection (p > 0.05).

**Table 3:** Treatment and follow-up of Stewart-Treves syndrome with upper limb lymphedema.

Case	Gender	Treatment	Follow-up
1	Female	Amputation	Survived without progress for 139 mos.
2	Female	Amputation	Died 48 mos.
3	Female	Amputation	Lung metastasis 2 mos. And died 6 mos.
4	Female	Amputation + chemotherapy	Died 26 mos.
5	Female	Chemotherapy	Died 7 mos.
6	Female	Radiotherapy + chemotherapy	Died 40 mos.
7	Female	Na	Died 3 mos.
8	Female	Na	Died 19 mos.
9	Female	Radiotherapy	Died 25 mos.
10	Female	Na	Died 35 mos.
11	Female	Na	Loss to follow-up
12	Female	Na	Loss to follow-up

**Table 4:** Treatment and follow-up of Stewart-Treves syndrome with lower limb lymphedema.

Case	Gender	Treatment	Follow-up
1	Female	Extended resection + chemotherapy	Died 31 mos.
2	Female	Extended resection + amputation	Survived for 72 mos. And pulmonary metastasis
3	Female	Extended resection	Died 11 mos.
4	Female	Extended resection + amputation + radiotherapy + chemotherapy	Survival for 41 mos., with lumbar and lung metastasis
5	Male	Extended resection	Died 2 mos.
6	Female	Extended resection	Died 3 mos.
7	Female	Na	Died 4 mos.
8	Male	Extended resection	Loss to follow-up
9	Female	Extended resection + amputation + radiotherapy	Died 26 mos.

## Discussion

Stewart-Treves syndrome is a rare angiosarcoma with a poor prognosis. It was first described by Stewart and Treves in 1948 in women with breast cancer treated with radical mastectomy.^7^ STS typically occurs as a complication of long-lasting lymphedema of the arm after axillary lymph node dissection following radical mastectomy and/or RT for breast cancer. STS can also appear in lower limbs with chronic lymphedema. It is essential to recognize suspicious lesions in a lymphedema limb early and to conduct a biopsy due to the high local recurrence rate, systemic dissemination, lack of a standardized treatment protocol and low survival rate within five years.^8^

STS are malignancies that originate from cells with endothelial function and the morphology of blood or lymphatic vessels. In the presence of lymphedema, they grow as plaques or cutaneous and subcutaneous nodules, single or multiple, which may coalesce, with an unknown etiology. Several studies have shown that lymphedema produces local immunosuppression as a result of lymphatic fluid stasis and the relative upregulation of growth factors within the microenvironment. This is thought to be associated with the potential development of malignant disease.^2^, ^9^ It is believed that deficiency in afferent lymphatic drainage prevents early recognition of tumor-specific antigens.^10^ When the local mechanisms of immune surveillance fail, the region becomes immunologically vulnerable and predisposed to cancer development. Some theories attempting to explain the mechanism have been proposed. Futrell and Myers highlighted the immunological status governing the response of animal hosts to skin-implanted tumors, with or without an intact lymphatic system. In their study, although the tumor solution failed to induce malignancy when injected into an area where the lymphatic vessels had been spared, large and lethal tumors developed whenever the injection was in an area where the lymphatics had been impaired.^11^ It is also believed that chronic stasis produces local changes in the lymphatic protein composition (decreased alpha-2 globulin fraction and increased albumin-globulin ratio) and that delaying protein transportation from the interstitial space into the lymphatic tissue might change the tissue antigenic composition and/or regional immunological competence. Lymphatic stasis and remodeling of connective tissue lead to local immunodeficiency. In addition, systemic immunodeficiency or systemic factors such as potential carcinogenic viral infections (human papillomavirus) also explain the etiology of tumors.^1^, ^12^

Previous studies and meta-analyses have reported that there are more women than men among patients with STS, the age of onset is generally middle-aged and elderly, and there are a very small number of adolescents under the age of 18. The related pathogenic factors of STS include high BMI (BMI ≥ 28), trauma history, limb disuse, radiotherapy history, malignancy history, and so on.^13^, ^14^, ^15^, ^16^ STS was most commonly reported on a unilateral upper extremity, and a few were on a lower extremity. The average time from limb swelling to malignancy ranges from a few months to several years, even decades. A few patients with lymphedema without a history of malignancy may last longer. Some studies have reported that microsurgery, immunotherapy, and drugs may be effective in STS treatment, but most treatment schemes still emphasize the importance of radical surgery in determining the survival rate of patients.^17^, ^18^, ^19^ In a study conducted in Canada, the average age of the patients was 61.2 years, and the average time from lymphedema to malignancy was 15.8 years. A total of 69.8% of the patients had a history of malignancy, and 53.7% had a history of radiotherapy.^20^ In a study by Stephen R. Grobmyer et al., there was no difference in the long-term survival rate between patients undergoing extensive resection and amputation, but the long-term survival rate of patients receiving chemotherapy and/or radiotherapy was lower.^21^ In another study, the prognosis was significantly improved after long-term follow-up for 6 patients who underwent amputation. Therefore, it is suggested that the most effective treatment for STS is radical ablative surgery, usually involving forequarter (or hindquarter) amputation.^22^, ^23^ In the retrospective analysis, the average age of 22 patients was 52.7 years, and most of them were over 35 years old. The average time from lymphedema to malignancy was 13 years. This is similar to the report in the literature. Furthermore, most STS patients had a history of malignancy, and the authors found that only amputation can improve the prognosis of patients. No difference in the survival rates between those patients treated with chemotherapy and those treated by radiotherapy has been found, which is consistent with some other studies.^21^, ^24^

Given the rising prevalence of lymphedema, physicians should be aware of the possibility of sarcomatous degeneration in lymphedema. There were 19 (86.4%) patients over 35 years old in the study, therefore, the occurrence of malignant tumors should be excluded when diagnosed with lymphedema tarda. The peak period for the diagnosis of STS is within six months and one year after lymphedema, suggesting that for patients with long-term chronic lymphedema, it is necessary to carry out close follow-up (three months, six months, one year after lymphedema) to identify malignant lesions as soon as possible. STS has an acute onset and a rapid progression, which results in changes in skin color. It is sometimes accompanied by pain, paraesthesia, paralysis, and weakness.^25^ Patients with sudden severe swelling or obvious aggravation of swelling in the short term need to pay special attention to the possibility of malignant lymphedema. Key components of the physical examination for patients with suspected STS should include the BMI value, distribution of edema, tenderness of the edema site, presence of pitting edema and varicose veins, any skin changes and any signs of systemic disease, including abdominal/pelvic masses or lymphadenopathies.^26^ Other signs also need special attention: whether there are swollen lymph nodes or masses in the inguinal or axillary region; whether there is cyanosis and venous reflux disorder (congestive changes of the surface veins) or nerve compression (with local pain or abnormal function of the affected limbs); whether there are changes in the skin appearance (rash, color change, necrosis or ulceration, etc.).^27^ In addition, an MRI scan is the standard procedure for the assessment of disease extension. When the imaging examination results were abnormal, inflammation of the lymphatic drainage area and tissue damage caused by surgery were excluded. As STS is a poorly differentiated neoplasm, it requires conﬁrmation by immunohistochemistry.^8^, ^28^ The disease demonstrates positivity for endothelial cell markers such as factor VIII and anti-CD34 and anti-CD31 antibodies, the latter being the one with greater sensitivity and speciﬁcity.^29^, ^30^

## Conclusion

Although the mechanism has yet to be illustrated, it is widely accepted that lymphedema may induce STS. Active diagnosis and treatment of lymphedema, paying close attention to the development of lymphedema, maintaining limb health and preventing infection are measures that can be adopted to avoid STS.^31^

## Financial support

Beijing Municipal Administration of Hospitals’ Youth Programme (QML20150101), Beijing Municipal Administration of Hospitals Incubating Program (PX2020030), Natural Science Foundation of Capital Medical University (PYZ2017158), Beijing Shijitan Hospital Fund Capital Medical University (2019-LB12).

## Authors’ contributions

Kun Hao: Approval of the final version of the manuscript; Critical literature review; Data collection, analysis and interpretation; Preparation and writing of the manuscript; Statistical analysis; Manuscript critical review.

Yuguang Sun: Approval of the final version of the manuscript; Critical literature review; Data collection, analysis and interpretation; Intellectual participation in propaedeutic and/or therapeutic management of studied cases.

Yan Zhu: Approval of the final version of the manuscript; Data collection, analysis and interpretation; Statistical analysis.

Jianfeng Xin: Approval of the final version of the manuscript; Data collection, analysis and interpretation.

Li Zhang: Approval of the final version of the manuscript; Data collection, analysis and interpretation.

Bin Li: Approval of the final version of the manuscript; Critical literature review; Data collection, analysis and interpretation; Effective participation in research orientation.

Wenbin Shen: Approval of the final version of the manuscript; Critical literature review; Data collection, analysis and interpretation; Intellectual participation in propaedeutic and/or therapeutic management of studied cases; Preparation and writing of the manuscript; Statistical analysis; Manuscript critical review.

## Conflict of interest

None declared.
